# Labels, Language, and Other Strategies to Improve Communication About Lower Grade Forms of Ductal Carcinoma In Situ of the Breast: A National Delphi Survey

**DOI:** 10.1155/ijbc/8642832

**Published:** 2025-02-17

**Authors:** Mavis S. Lyons, Genevieve Chaput, Antonio Finelli, Rachel Kupets, Nicole Look Hong, Frances C. Wright, Anna R. Gagliardi

**Affiliations:** ^1^Toronto General Hospital Research Institute, University Health Network, Toronto, Ontario, Canada; ^2^McGill University Health Centre, McGill University, Montreal, Quebec, Canada; ^3^Princess Margaret Cancer Centre, University Health Network, Toronto, Ontario, Canada; ^4^Odette Cancer Centre, Sunnybrook Health Sciences Centre, Toronto, Ontario, Canada

**Keywords:** communication, Delphi survey, ductal carcinoma in situ, person-centered care

## Abstract

**Purpose:** This study is aimed at generating consensus among women who had ductal carcinoma in situ (DCIS) and healthcare professionals on how to improve communication about low-risk forms of DCIS and reduce affected women's diagnosis-related confusion and anxiety.

**Methods:** We conducted a two-round online Delphi survey with affected women and professionals from across Canada. They rated items sourced from prior research and key informant interviews on a 7-point Likert scale. We retained items rated 6 or 7 by ≥ 80% of panelists.

**Results:** Thirty-seven panelists (17 women, 20 professionals) completed Round 1 and 94.6% of those completed Round 2. Of 42 items rated, 18 were retained, 13 discarded, and 11 did not achieve consensus to retain or discard. Women and professionals agreed on 3 language approaches (use plain language, distinguish DCIS from invasive breast cancer, specify the risk of recurrence and spread) and 9 other strategies to help discuss DCIS (e.g., use visual aids, provide or refer women to culturally tailored DCIS-specific information, ensure physicians can access interpreters). Based on rating and comments, women were more enthusiastic than professionals about referring to abnormal cells rather than DCIS and scheduling longer or follow-up visits to address concerns. To disseminate these findings, panelists recommended public awareness campaigns for women and continuing education and professional society endorsement for physicians.

**Conclusion:** These findings address gaps in prior research that recommended changing the DCIS label, but had not fully explored label preferences, or identified other ways to improve and support communication about DCIS.

## 1. Introduction

Due to cancer screening, many people are diagnosed with “low-risk” lesions that may remain indolent. Without a standard definition, in this work, low risk refers to lesions that are unlikely to progress to invasive cancer or, if treated, are unlikely to recur. Ductal carcinoma in situ (DCIS) of the breast is an example of such a lesion. DCIS refers to a spectrum of abnormal cells confined to the breast ducts that comprises 15%–25% of screen-detected breast lesions [1]. Approximately 20% of DCIS cases evolve to invasive breast cancer, with a low 20-year breast-cancer specific mortality of 3.3% [2, 3]. While not yet established, low-risk DCIS (henceforth, DCIS) may include low and intermediate nuclear grade [4]. Until ongoing trials or future research definitively shows that active surveillance is a safe option for low-risk forms of DCIS, guideline-recommended management involves treatment with lumpectomy or mastectomy +/− radiotherapy and/or hormone therapy [5].

Considerable prior research revealed challenges in communication about DCIS in general. Women with DCIS from many countries have reported that physicians provided scant or unclear information about DCIS, causing confusion and anxiety, which persisted years after treatment, leading to poor physical and psychological outcomes and reduced quality of life [6, 7]. Physicians from the United Kingdom and United States reported challenges in distinguishing DCIS from cancer [8, 9]. More recently, focus groups with diverse women who had DCIS revealed that they were frustrated by the confusing terminology, lack of information about the benefits of treatment, and risk of progression or recurrence [10]. Interviews with diverse physicians revealed variations in whether they referred to DCIS as cancer and confirmed difficulty in justifying treatment while simultaneously assuring women of a good prognosis [11].

Clearly, efforts are needed to improve communication about DCIS, particularly for lower-grade forms with a low risk of progressing to invasive cancer. A review of prior research on how to improve DCIS communication identified no studies that developed or evaluated such interventions aimed at women or physicians [12]. As a first step, women with DCIS and physicians of various specialties from across Canada recommended identifying noncancer labels and language to discuss DCIS acceptable to both affected women and physicians [13] Women diagnosed with low-risk forms of DCIS are suffering from needless confusion and anxiety that could potentially be alleviated through improved communication. The overall aim of this research was to generate recommendations for improving DCIS communication that could be widely promoted and adopted by advocacy groups, professional societies, and individual physicians, which may reduce confusion and anxiety among affected women. The specific objective of this study was to establish the consensus of women who had DCIS and healthcare professionals on the ideal labels, language, and other strategies to discuss DCIS.

## 2. Methods

### 2.1. Approach

We conducted a Delphi survey, a widely used approach to establish consensus on healthcare recommendations [14], and complied with the Conducting and REporting of DElphi Studies criteria [15]. The Delphi technique relies on response frequency to generate consensus through repeat surveys. Round #1 identified agreement to retain or discard items, and Round #2 generates agreement to retain or discard items that did not achieve consensus in Round #1. Two survey rounds are proven to prevent respondent fatigue and dropout [16, 17].

### 2.2. Sampling and Recruitment

While the historical median number of panelists is 17, larger panel sizes were found to enhance reliability and better accommodate diversity [16, 17]. Therefore, to increase reliability and to best capture the perspectives of diverse women and healthcare professionals, we used maximum variation sampling to recruit a 30-member panel from across Canada, with 50% representing women with a history of DCIS. We recruited women aged 18+ who had DCIS in the past 15 years from across Canada. Women were recruited through collaborating agencies, coinvestigators, breast cancer advocacy groups, and immigrant settlement agencies that provided health promotion services. We aimed to recruit physicians who counseled, diagnosed, or treated women with DCIS (e.g., family physicians, general surgeons; surgical, medical, or radiation oncologists; pathologists) and other professionals with insight on cancer screening or communication (e.g., healthcare system managers, researchers) through collaborating agencies, coinvestigators, and professional societies and via the websites of medical school faculty across Canada. Potential participants were instructed to email the study team to express interest, and others were contacted directly by email. All participants provided written informed consent.

### 2.3. Survey Development

To inform survey items, we reviewed prior research on preferred labels for DCIS [18] and for other types of low-risk lesions [19] and interviewed key informants including women with DCIS and physicians who care for them [20] and patients with other types of low-risk lesions (cervix, bladder, prostate) and physicians who care for them [21]. We included other types of low-risk lesions to gather any insight on ideal communication approaches. From this work, we compiled unique survey items into four categories: (1) preferred labels for DCIS, (2) language to explain DCIS, (3) other strategies to help explain DCIS, and (4) dissemination approaches to promote awareness and use of labels, language, and other strategies. Supporting Information 1: File S1 reports the source of survey items. The survey was administered in REDCap and prompted panelists to rate the importance of each item on a 7-point Likert scale (1 *strongly disagree*, 7 *strongly agree*). For each category, panelists could enter comments to explain their rating and, in Round #1 only, could suggest labels, language, or other strategies not already included in the survey.

### 2.4. Data Collection and Analysis

The Round #1 survey link was shared with consenting participants on June 3, 2024, with reminders at 2, 3, and 4 weeks. A Round #1 report summarizing rating frequencies and comments was shared with panelists, inviting them to review the Round #1 report and complete the Round #2 survey. The Round #1 report organized items as those retained (≥ 80% of panelists rated 6 or 7), those discarded (≥ 80% of panelists rated 1 or 2), and those that did not achieve consensus to retain or discard. The Round #2 survey included only the items that did not achieve consensus to retain or discard. The Round #1 report and Round #2 survey link were shared with participants on September 3, 2024, with reminders to complete the Round #2 survey at 2, 3, and 4 weeks. We analyzed and summarized Round #2 results similarly to Round #1 and reported results overall and by subgroup (i.e., women, professionals).

### 2.5. Patient and Public Involvement

The research team included women who had been treated for DCIS of diverse age, ethnocultural group, DCIS treatment, and region of Canada. They were routinely engaged in study conception and planning, data collection and analysis, and manuscript writing.

## 3. Results

### 3.1. Participants

Table 1 reports participant characteristics. Of the 17 women, most were aged 51–66 (64.7%), resided in the province of Ontario (76.5%), and were White (58.8%). Time since diagnosis ranged from less than 1 year to 13 years, and a nearly equal number of women had surgery only or surgery and adjuvant therapy. Of the 20 professionals, most were in the self-reported late stage of their career (45.0%), surgical oncologists (35.0%), and women (65.0%). Professionals resided in seven Canadian provinces. The Round #2 response rate was 94.1% (16/17) for women and 95.0% (19/20) for professionals or 94.6% overall.

### 3.2. Overall Delphi Results

**Figure 1:**
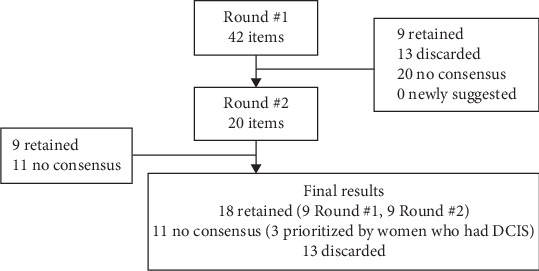
Overall Delphi results.

Supporting Information 2: File S2 details the strategies that panelists agreed to retain or discard in Round #1 and Round #2.  Figure 1 summarizes the number of items retained, discarded, or with no consensus in each round. Overall, of the initial 42 items, 18 achieved consensus to retain: 9 in Round #1 and 9 in Round #2.

### 3.3. Delphi Results by Category and Group

Table 2 reports items that achieved consensus to retain. Supporting Information 2: File S2 provides ratings for all items retained, discarded, or that did not achieve consensus, which yields insight on level of agreement between women and professionals. Supporting Information 3: File S3 provides all comments offered by panelists, which yield insight on why some items were not retained.

#### 3.3.1. Labels for DCIS

With respect to labels for DCIS, none of the 14 options were recommended. This was true for all options categorized as referring to abnormal cells, precursor to cancer, or cancer (including DCIS). Woman and professional views about labels for DCIS were closely aligned, with no labels achieving consensus to retain. Mixed views were expressed within and between groups. Labels referring to abnormal cells were considered by some to be too vague, and precursor and cancer labels were not favored because they were unclear and inaccurate and mentioned cancer (including DCIS).

Abnormal label

Abnormal cells could be anything and could give patients a false sense of security. (Woman)

Too vague and do not carry the gravity that is needed. (Professional)

Precursor label

Neoplasia and dysplasia do not mean anything to me so if those words were in the name I may not understand what that meant. (Woman)

The cells are not dysplasia nor atypia, so we should not use those words. (Professional)

Cancer label

I notice that most of these still use the word cancer. (Woman)

Any label with “cancer” in the wording will spark concern in patients. (Professional)

The only label that came close to achieving consensus was “abnormal cells of the breast duct that have not spread to breast tissue outside of ducts,” which was rated by 81.3% of women panelists as favorable compared with 73.7% of professionals.

#### 3.3.2. Language to Explain DCIS

Three items achieved consensus to retain: use plain language rather than medical terminology to explain DCIS, explicitly state that DCIS is not the same as invasive breast cancer, and specify the risk of reoccurrence and spread and the likely prognosis of low-risk forms of DCIS. Woman and professional rating for using plain language was aligned (88.2% and 90.0%, respectively), but women were more enthusiastic than professionals about distinguishing DCIS from invasive breast cancer (87.5% vs. 73.7%, respectively) and specifying risk and prognosis (94.0% vs. 75.0%, respectively). Comments only from professionals revealed the need to refine the wording of these two items.

State that DCIS is not invasive breast cancer because it IS CURRENTLY in the breast duct. (Professional)

Currently there is no way to know what DCIS is “going to do” in any given patient case so I do not endorse that language. (Professional)

For items that did not achieve consensus, woman and professional ratings were similar for using analogies, stating that DCIS is common and describing DCIS as a spectrum of types of cells where some may not require treatment. Comments revealed that both groups thought these items were confusing, vague, and inaccurate.

[Analogies] may lead to further confusion, especially if English not first language. (Professional)

Does it matter that DCIS is common? Unless there is context, I do not know if this is helpful. (Woman)

#### 3.3.3. Strategies to Help Explain DCIS

Nine items achieved consensus to retain, with the highest priority being develop culturally tailored DCIS-specific informational resources, provide or refer women to informational resources about DCIS, and ensure that physicians have access to interpreters for women with English as a second language. Other items that achieved consensus included use of visual aids, refer to pathology or radiology reports during discussion, ask women to express specific concerns, and connect women with support services or groups. Woman and professional ratings were aligned for use of visual aids and interpreters, providing or referring women to print or online resources, and developing culturally tailored DCIS-specific resources. Women were more enthusiastic than professionals for use of pathology or radiology reports (87.5% vs. 73.7%, respectively), asking women about specific concerns (100.0% vs. 70.0%, respectively), and connecting women with support services or groups (93.0% vs. 68.4%, respectively).

For items that did not achieve consensus, woman and professional ratings were similar for sending women news about a DCIS diagnosis before for the first physician visit as this would prompt anxiety among women. Woman and professional ratings were not aligned for schedule longer physician visits to discuss concerns (100.0% vs. 52.6%, respectively) or arrange follow-up visits to discuss further concerns or questions (81.3% vs. 57.9%, respectively).

Extra time may be difficult to manage logistically in today's health care system. (Professional)

For the single discarded item about being cared for in the community rather than a designated cancer center, woman and professional ratings and comments were similar. Both groups commented that it was best to address DCIS in a center that could provide specialized information and support.

Being treated in a cancer centre might give the person with DCIS a sense of comfort, as in, I am being treated by those who know cancer. (Woman)

#### 3.3.4. Dissemination of Results

Panelists achieved consensus to retain six items, with the highest priority being share information about DCIS via existing breast cancer public awareness campaigns, share information with physicians to encourage using the language and other strategies to explain DCIS, professional societies should share this information with physicians and also offer continuing education opportunities about DCIS, publish these findings in a medical journal, and establish multidisciplinary collaboration among advocacy and professional organizations to promote use of these findings. Woman and professional ratings and comments were largely aligned.

The single item that was discarded, share the results of this research with nomenclature agencies to potentially influence the naming of low-risk DCIS, was rated highly by women (87.5%) but not professionals (63.2%), who thought it best to instead focus on using the language and other strategies identified in this study to improve communication about DCIS.

Let us not rebrand DCIS but just provide more specific educational resources. (Professional)

A single item was discarded: change DCIS labels and language in medical records, which are now accessible to patients. Rating was similarly low for both women and professionals, but only professionals commented.

Without this, the research which ultimately informs risk and permits de-escalation or escalation of treatment will be hampered. (Professional)

## 4. Discussion

In this national-level Delphi study, 37 panelists (17 women and 20 professionals) established consensus on 3 language approaches and 9 other strategies to improve discussions about lower-grade forms of DCIS with a low risk of progressing to invasive cancer and, apart from this publication, 6 dissemination options to promote use of these findings. For some items that did not achieve consensus, women were more enthusiastic than professionals, which identified recommendations important to women with DCIS (referring to DCIS as abnormal cells, taking extra time or scheduling longer visits to discuss concerns, and/or arranging follow-up visits to address remaining concerns).

To contextualize these findings, some prior research identified that women preferred the term abnormal cells over a term such as DCIS that referred to cancer [22, 23]. However, those studies involved women and not physicians or other professionals, the women had no personal history of breast cancer or DCIS, and study objectives largely focused on views about management of DCIS. For indolent lesions of the cervix, bladder, and prostate, nomenclature was officially changed to eliminate cancer terms, which sets a precedence, but little research or engagement of affected patients preceded those efforts to yield insight on motivation for the nomenclature changes, and little subsequent research examined the impact of nomenclature changes on either patients or professionals [24, 25]. A 2024 review of published research on preferred labels and language for low-risk cervix, bladder, prostate, and thyroid lesions identified a paucity of research [19]. However, three studies suggested that modified labels lacked meaning to patients, who were not clear whether they had cancer or not, why they required treatment, and what it meant for their future health [26–28]. Thus, even if nomenclature omitted cancer-related terms, there remains a need for accompanying language or other strategies that better explain the implications of low-risk lesions. Overall, this study fills a void in prior research that did not fully explore the DCIS label preferences of women and professionals or identify other ways to improve and support communication about DCIS from the perspective of both women who had DCIS and professionals.

These findings raise several implications for future practice and research. While the overall panel did not achieve consensus for an alternative label to DCIS, women prioritized referring to DCIS as abnormal cells. As noted in panelist comments, physicians may continue to refer to DCIS, plus women with access to their medical records may see DCIS in radiology or pathology reports. However, it may be a useful approach to explain DCIS by referring to abnormal cells, as has also been revealed in prior research [22, 23].

Panelists recommended three language approaches to describe DCIS. To widely encourage use of such language by physicians, panelists recommended continuing education for physicians about DCIS and that professional societies endorse these findings. Another way to share information with physicians is via clinical practice guidelines that are often developed by professional societies [29]. Research shows that professional societies are deeply and regularly engaged with their members and play a critical role in influencing health system transformations [30].

Panelists recommended nine other strategies to help explain DCIS (visual aids, provide/refer women to DCIS resources, provide interpreters). Prior research revealed challenges with the accuracy and impact of interpreters for those with English as a second language in the context of cancer. For example, misinterpretation can occur via both professional and family interpreters [31, 32]. Therefore, future research should identify strategies that can optimize the accessibility and accuracy of interpreters, particularly in the DCIS context. While panelists largely agreed on other strategies, women were more enthusiastic than professionals for some strategies. To ensure that women's preferences are addressed, it will be important to underscore the differing views of women and professionals revealed in this research when disseminating the findings. Also, even though professional panelists said that longer or follow-up visits are not feasible, research shows that nonphysician members of interdisciplinary cancer teams can play an important role in addressing questions or concerns [33]; and cancer patients receiving interdisciplinary treatment reported almost four times more positive perceptions of communication compared with noninterdisciplinary care [34].

Instead of trying to influence nomenclature agencies, panelists thought it more important to focus dissemination efforts on encouraging physicians to use the language and other strategies generated by this research. Clinicians convened at a 2010 meeting of the American Cancer Society-National Cancer Institute and a 2011 World Health Organization meeting decided against renaming DCIS due to a lack of evidence on predictors of progression to invasive cancer [35, 36]. Despite advocates first recommending DCIS be renamed in 1998 [37] and a plethora of evidence on harms caused by poor DCIS communication [6, 7, 10, 38, 39], no action has been taken. Until ongoing trials generate definitive evidence on optimal management of low-risk forms of DCIS [40–42], nomenclature agencies may not consider renaming DCIS; however, this study revealed that neither women nor professionals believe that DCIS is a suitable label.

With respect to ongoing research, panelists recommended developing culturally tailored DCIS-specific informational resources. Our analysis of 39 online resources revealed that they largely failed to distinguish DCIS from invasive breast cancer [43]. Some research has already revealed the questions and issues of most concern to women with DCIS [10, 44], but additional research that engages ethnoculturally diverse women is needed to ensure such information is culturally relevant and identify how to best share such information with diverse women. For example, community agencies such as cultural groups and immigrant settlement services appear to play an important role in health promotion to diverse women [45, 46]. Future research could compare comprehension, anxiety, and health-related quality of life between women diagnosed with DCIS exposed and not exposed to the language and strategies recommended by panelists as a way to validate the study. Also, researchers might investigate the impact of language and other strategies revealed in this study on people diagnosed with abnormal cells of the cervix, bladder, and prostate [26–28].

This study featured several strengths. We employed rigorous methods and complied with research standards for conducting and reporting Delphi studies [14–17]. We actively engaged ethnoculturally diverse women and an interdisciplinary research team in planning and executing this study. We identified strategies from multiple sources [18–21]. Strategies were rated by a larger number of respondents than is typical of most Delphi studies to ensure that participants reflected varied perspectives and because larger sample size has been shown to enhance reliability [17]. Furthermore, we applied a stringent definition of consensus but also reported items for which women were more enthusiastic than professionals. We acknowledge potential limitations. As research volunteers, the participants may have a particular interest in DCIS; thus, their views may be biased. Two women were diagnosed with DCIS 13 years ago, and their perspectives may have been influenced by recall bias. Women and professionals did not provide written comments for each survey item, potentially limiting insight on ratings; however, all survey items were informed by prior research that captured the in-depth views of women and professionals [18–21]. The prioritized strategies may not be relevant to those from other disadvantaged groups or to women or professionals outside of Canada with differing health systems.

## 5. Conclusions

A total of 37 panelists (17 women and 20 professionals) agreed on 3 language approaches and 9 other strategies to help explain lower-grade forms of DCIS with a low risk of progressing to invasive cancer along with 6 dissemination options to encourage use of recommended language and other strategies. By comparing rating and comments, we also identified that women were more enthusiastic than professionals about referring to DCIS as abnormal cells and about the need for longer and/or follow-up visits to address women's concerns and questions. These findings address gaps in prior research that recommended changing the DCIS label, but had not fully explored label preferences or rationale among both women and professionals, or identified other ways to improve and support communication about DCIS. Clearly, changing the DCIS label alone is insufficient, and language and other strategies to improve communication about DCIS should be consistently applied. This knowledge could be endorsed and widely shared by advocacy groups and professional societies with physicians so that they employ language and other strategies that may reduce confusion and anxiety among women diagnosed with DCIS.

## Figures and Tables

**Table 1:** Panelist characteristics.

**Group**	**Characteristic**	**Category**	**n** ** (%)** **Round #1**
Women	Age (years)	≤ 50	6 (35.3)
		51–66	11 (64.7)

Round #1 *n* = 17	Time since diagnosis (years)	< 1	6 (35.3)
		1–4	5 (29.4)
		5–13	6 (35.3)

Round #2 *n* = 16	Province in Canada	Ontario	13 (76.5)
		British Columbia	2 (11.8)
		Alberta	1 (5.9)
		Saskatchewan	1 (5.9)
	Ethnocultural group	Caucasian/White	10 (58.8)
		African or Caribbean Black	3 (17.6)
		South Asian	2 (11.8)
		Middle Eastern	1 (5.9)
		East Asian	1 (5.9)
	Treatment received	Surgery + adjuvant therapy	9 (52.9)
		Surgery only	8 (47.1)

Professionals	Career stage	Early	3 (15.0)
		Mid	8 (40.0)
		Late	9 (45.0)

Round #1 *n* = 20	Specialty	Surgical oncologist	7 (35.0)
		Radiation oncologist	4 (20.0)
		Family physician	3 (15.0)
		Pathologist	2 (10.0)
		General surgeon	1 (5.0)
		Medical oncologist	1 (5.0)
		Screening manager	1 (5.0)
		Researcher	1 (5.0)

Round #2 *n* = 19	Province in Canada	British Columbia	6 (30.0)
		Ontario	6 (30.0)
		Nova Scotia	3 (15.0)
		Quebec	2 (10.0)
		Manitoba	1 (5.0)
		New Brunswick	1 (5.0)
		Saskatchewan	1 (5.0)
	Gender	Woman	13 (65.0)
		Man	7 (35.0)

**Table 2:** Delphi items that achieved consensus to retain.

**Category (** **n** ** of total items retained)**	**Item**	**n** ** (%) of panelists that rated items 6 or 7 on the Likert scale**		**Total ** **n** ** (%)**
		**Women**	**Professionals**	
Preferred labels for DCIS (0/14)	—	—	—	—

Language to explain DCIS (3/7)	Use plain/lay language that patients will understand to explain DCIS	15 (88.2)	18 (90.0)	33 (89.2)
	Address risks (e.g., spread, recurrence) and outcomes (e.g., prognosis) associated with low-risk DCIS	16 (94.1)	15 (75.0)	31 (83.8)
	State that DCIS is not invasive breast cancer because it stays in the breast duct and is unlikely to spread	14 (87.5)	14 (73.7)	28 (80.0)

Strategies to help explain DCIS (9/13)	Give physicians access to interpreters for patients with English as a second language	15 (88.2)	18 (90.0)	33 (89.2)
	Provide patients with or refer them to print or online resources about DCIS	15 (93.8)	16 (84.2)	31 (88.6)
	Develop information for patients that is specific to DCIS (not included in resources about invasive breast cancer)	16 (94.1)	16 (80.0)	32 (86.5)
	Use visual aids (pictures, models) to help explain DCIS	15 (88.2)	16 (80.0)	31 (83.8)
	Provide physicians with visual aids or guides to help explain DCIS	14 (82.4)	17 (85.0)	31 (83.8)
	Ask patients about specific concerns	17 (100.0)	14 (70.0)	31 (83.8)
	Develop information for patients about DCIS that is culturally tailored (e.g., available in different languages)	15 (88.2)	16 (80.0)	31 (83.8)
	Connect patients with services or groups for more information and support	15 (93.8)	13 (68.4)	28 (80.0)
	Use pathology or radiology report to supplement discussion	14 (87.5)	14 (73.7)	28 (80.0)

Recommended strategies to disseminate the results of this research (6/8)	Existing breast cancer public awareness campaigns and support groups should share information with women about DCIS	16 (100.0)	17 (89.5)	33 (94.3)
	Professional societies should share information with physicians about ideal labels, language, and other strategies to improve communication about DCIS	16 (100.0)	16 (84.2)	32 (91.4)
	Various types of organizations should provide continuing education for physicians (meetings and materials) about DCIS	16 (100.0)	15 (78.9)	31 (88.6)
	Physicians should employ labels, language, and other strategies identified by this research to decrease patient anxiety	16 (94.1)	16 (80.0)	32 (86.5)
	Publish the results of this research in a prominent medical journal to encourage widespread use of ideal labels, language, and other strategies	15 (93.8)	14 (73.7)	29 (82.9)
	Organizations (e.g., professional, advocacy) should collaborate to establish widespread multidisciplinary consensus on ideal DCIS labels, language, and other strategies to improve communication about DCIS	14 (87.5)	15 (78.9)	29 (82.9)

## Data Availability

The datasets generated during and/or analyzed during the current study are available in the manuscript and its supporting information.

## Supporting Information

Supporting Information 1 File S1: Source of Delphi survey items.

Supporting Information 2 File S2: Delphi results.

Supporting Information 3 File S3: Panelist comments about survey items.
