# Supplementary material for: Labels, Language, and Other Strategies to Improve Communication About Lower Grade Forms of Ductal Carcinoma In Situ of the Breast: A National Delphi Survey
Source: Int J Breast Cancer. 2025 Feb 17;2025:8642832. doi: 10.1155/ijbc/8642832 (PMC11850068; doi:10.1155/ijbc/8642832)
Supplement: Supporting Information 1 — File S1: Source of Delphi survey items. [file 8642832.f1.docx]

**Supplementary File 1. Source of Delphi survey items**

SECTION 1. Preferred label for DCIS

- People with DCIS, bladder, cervix and prostate lesions preferred “abnormal cells”, especially when first diagnosed, because it was easy to understand, and prompted less anxiety than precursor to cancer (e.g. stage 0 breast cancer) or cancer-related (e.g. non-invasive breast cancer) labels
- Physicians who care for people with DCIS, bladder, cervix and prostate cancer preferred precursor to cancer and cancer-related labels because they assumed patients understood such labels implied low risk for cancer progression and would not be worried, and to convince patients of the need for treatment or follow-up visits

| Item | Review of prior research | DCIS interviews | | Bladder, cervix, prostate interviews | |
| --- | --- | --- | --- | --- | --- |
|  |  | Women | Physicians | Patients | Physicians |
| Abnormal cells | | | | | |
| Abnormal cells of the breast duct | X | X |  | X | X |
| Atypical cells of the breast duct |  |  |  |  | X |
| Breast duct dysplasia |  |  |  |  | X |
| Precursor to cancer cells | | | | | |
| Pre-cancer | X | X | X | X | X |
| Stage 0 breast cancer | X | X | X |  |  |
| Pre-invasive breast cancer | X | X | X |  |  |
| Cancer cells | | | | | |
| Early-stage breast cancer | X |  |  |  |  |
| Ductal carcinoma *in situ* |  |  |  |  | X |
| Low-risk/Low-grade breast cancer |  |  |  |  | X |
| Non-aggressive breast cancer |  |  |  |  | X |
| Breast duct neoplasia |  |  | X |  |  |
| Non-invasive breast cancer |  |  | X |  |  |
| Early form of breast cancer | X | X | X |  |  |

SECTION 2. Language to explain DCIS

- People with DCIS, bladder, cervix and prostate lesions said that their physician did not explain the meaning of precursor to cancer or cancer-related labels, or explained their diagnosis using medical/technical language
- Physicians who care for people with DCIS, bladder, cervix, and prostate lesions said they explained the diagnosis by noting it was not cancer or at the low end of the spectrum of cancer cells, and unlikely to spread

| Item | Review of prior research | DCIS interviews | | Bladder, cervix, prostate interviews | |
| --- | --- | --- | --- | --- | --- |
|  |  | Women | Physicians | Patients | Physicians |
| Use plain/lay language to explain DCIS | X | X | X | X | X |
| State that DCIS is not invasive breast cancer because it stays in the breast duct and is unlikely to spread | X | X | X |  | X |
| Use analogies to explain DCIS |  |  |  |  | X |
| Mention that DCIS is very common |  |  |  | X | X |
| Explain DCIS as a spectrum of cells, where some forms require treatment and other low-risk forms may not |  |  | X |  | X |
| Address risks (e.g. spread, recurrence) and outcomes (e.g. prognosis) for low-risk DCIS | X | X |  |  |  |
| Discuss risk based on stage or grade to explain why treatment is suggested for low-risk DCIS |  |  | X |  |  |

SECTION 3. Other strategies to help explain DCIS

- People with DCIS, bladder, cervix, and prostate lesions said that their physicians did not use other strategies to help explain their diagnosis, and had to do their own searching for more information about their diagnosis
- Physicians who care for people with DCIS, bladder, cervix, and prostate lesions said they used visual aids, took extra time to answer questions and provided take-home information

| Item | Review of prior research | DCIS interviews | | Bladder, cervix, prostate interviews | |
| --- | --- | --- | --- | --- | --- |
|  |  | Women | Physicians | Patients | Physicians |
| Send patients information about their diagnosis of DCIS before the first physician visit so they can prepare questions |  |  |  |  | X |
| Take extra time or schedule longer visits to discuss concerns and answer questions |  | X | X | X | X |
| Ask patients about specific concerns |  |  |  |  | X |
| Use visual aids (pictures, models) to help explain DCIS |  |  | X | X | X |
| Use pathology or radiology reports to supplement discussion |  |  |  |  | X |
| Provide physicians with visual aids or guides to help explain DCIS |  |  | X | X | X |
| Give physicians access to interpreters for patients with English as a second language | X |  |  |  |  |
| Provide patients with, or refer them to print or online resources about DCIS |  | X | X | X | X |
| Connect patients with services or groups for more information and support |  |  |  | X | X |
| Develop information for patients that is specific to DCIS (not included in resources about invasive breast cancer) | X |  |  |  |  |
| Develop information for patients about DCIS that is culturally tailored (e.g. available in different languages) | X |  |  |  |  |
| Arrange follow-up visit not long after first visit to discuss further concerns/ questions |  |  |  |  | X |
| Do not manage low-risk DCIS in cancer centres to avoid giving patients the idea that they have full-blown cancer |  |  | X |  |  |

SECTION 4. Recommendations to disseminate findings of this research

People with DCIS, bladder, cervix, and prostate lesions and physicians who care for them agreed on several ways to improve communication about DCIS, and encourage use of ideal labels, language and other strategies

| Strategy | Review of prior research | DCIS interviews | |
| --- | --- | --- | --- |
|  |  | Women | Physicians |
| Physicians should employ labels, language and other strategies identified by this research to decrease patient anxiety | X | X | X |
| Existing breast cancer public awareness campaigns and support groups should share information with women about DCIS |  | X | X |
| Various types of organizations should provide continuing education for physicians (meetings and materials) about DCIS |  | X | X |
| Professional societies should share information with physicians about ideal labels, language and other strategies to improve communication about DCIS |  |  | X |
| Organizations (e.g. professional, advocacy) should collaborate to establish widespread multidisciplinary consensus on ideal DCIS labels, language and other strategies to improve communication about DCIS |  | X | X |
| Change DCIS labels and language currently in medical records (now accessible to patients) to those identified in this research | X |  | X |
| Share the results of this research with cancer nomenclature agencies, which may influence processes and decisions about naming for low-risk DCIS |  |  | X |
| Publish the results of this research in a prominent medical journal to encourage widespread use of ideal labels, language and other strategies | X |  | X |
