# Supplementary material for: Labels, Language, and Other Strategies to Improve Communication About Lower Grade Forms of Ductal Carcinoma In Situ of the Breast: A National Delphi Survey
Source: Int J Breast Cancer. 2025 Feb 17;2025:8642832. doi: 10.1155/ijbc/8642832 (PMC11850068; doi:10.1155/ijbc/8642832)
Supplement: Supporting Information 2 — File S2: Delphi results. [file 8642832.f2.docx]

**Supplementary File2. Delphi results**

Preferred label(s) for DCIS

| Survey item | Label | Revision suggested in Round #1 and offered in Round #2 | Rating (% panelists who chose 6 or 7 on the 7-point Likert scale) | | | | Decision |
| --- | --- | --- | --- | --- | --- | --- | --- |
|  |  |  | Round #1 | | Round #2 | |  |
|  |  |  | Women | Clinicians | Women | Clinicians |  |
| 1 | Abnormal cells of the breast duct | Abnormal cells of the breast duct that have not spread to breast tissue outside of ducts | No consensus  (64.7) | No consensus  (50.0) | Retain  (81.3) | No consensus (73.7) | No consensus |
| 2 | Atypical cells of the breast duct | Not favoured by panelists; not included in Round #2 survey | No consensus  (41.2) | No consensus  (25.0) | --- | --- | Discard |
| 3 | Breast duct dysplasia | Not favoured by panelists; not included in Round #2 survey | No consensus  (0.0) | No consensus  (10.0) | --- | --- | Discard |
| 4 | Pre-cancer | None | No consensus  (58.8) | No consensus  (50.0) | No consensus (43.8) | No consensus (63.2) | No consensus |
| 5 | Stage 0 breast cancer | None | No consensus  (41.2) | No consensus  (35.0) | No consensus (75.0) | No consensus (36.8) | No consensus |
| 6 | Pre-invasive breast cancer | Not favoured by panelists; not included in Round #2 survey | No consensus  (29.4) | No consensus  (20.0) | --- | --- | Discard |
| 7 | Early-stage breast cancer | Not favoured by panelists; not included in Round #2 survey | No consensus  (35.3) | No consensus  (5.0) | --- | --- | Discard |
| 8 | Ductal carcinoma in situ | None | No consensus  (35.3) | No consensus  (45.0) | No consensus (50.0) | No consensus (36.8) | No consensus |
| 9 | Low-risk breast cancer | Not favoured by panelists; not included in Round #2 survey | No consensus  (35.3) | No consensus  (15.0) | --- | --- | Discard |
| 10 | Low-grade breast cancer | Not favoured by panelists; not included in Round #2 survey | No consensus  (35.3) | Discard  (5.0) | --- | --- | Discard |
| 11 | Non-aggressive breast cancer | Not favoured by panelists; not included in Round #2 survey | No consensus  (23.5) | No consensus  (0.0) | --- | --- | Discard |
| 12 | Breast duct neoplasia | Not favoured by panelists; not included in Round #2 survey | No consensus  (17.6) | No consensus  (5.0) | --- | --- | Discard |
| 13 | Non-invasive breast cancer | Not favoured by panelists; not included in Round #2 survey | No consensus  (17.6) | No consensus  (10.0) | --- | --- | Discard |
| 14 | Early form of breast cancer | Not favoured by panelists; not included in Round #2 survey | No consensus  (17.6) | No consensus  (5.0) | --- | --- | Discard |

Language to explain DCIS

| Survey item | Label | Revision suggested in Round #1 and offered in Round #2 | Rating (% panelists who chose 6 or 7 on the 7-point Likert scale) | | | | Decision |
| --- | --- | --- | --- | --- | --- | --- | --- |
|  |  |  | Round #1 | | Round #1 | |  |
|  |  |  | Women | Clinicians | Women | Clinicians |  |
| 15 | Use plain/lay language to explain DCIS | Use plain/lay language that patients will understandto explain DCIS | Retain  (88.2) | Retain  (90.0) | --- | --- | Retain |
| 16 | State that DCIS is not invasive breast cancer because it stays in the breast duct and is unlikely to spread | None | No consensus (76.5) | No consensus (75.0) | Retain  (87.5) | No consensus (73.7) | Retain  [combined rating of 80.0] |
| 17 | Use analogies to explain DCIS | None | No consensus (52.9) | No consensus (45.0) | No consensus (62.5) | No consensus (26.3) | No consensus |
| 18 | Mention that DCIS is very common | Mention that DCIS affects many women | No consensus (52.9) | No consensus (20.0) | No consensus (50.0) | No consensus (21.1) | No consensus |
| 19 | Explain DCIS as a spectrum of cells, where some forms require treatment and low-risk forms may not | Explain DCIS by grade, where higher-grade forms of DCIS require treatment, and low-grade forms may not | No consensus (52.9) | No consensus (20.0) | No consensus (56.3) | No consensus (15.8) | No consensus |
| 20 | Address risks (e.g. spread, recurrence) and outcomes (e.g. prognosis) associated with low-risk DCIS | None | Retain  (94.1) | No consensus (75.0) | --- | --- | Retain  [combined rating of 83.8] |
| 21 | Discuss risk based on stage or grade to explain why treatment is recommended for low-risk DCIS | Similar to previous item so this item was not included in Round #2 survey, and was discarded | Retain  (88.2) | No consensus (50.0) | Not included in Round #2 survey | Not included in Round #2 survey | Discard |

Other strategies to help explain DCIS

| Survey item | Label | Revision suggested in Round #1 and offered in Round #2 | Rating (% panelists who chose 6 or 7 on the 7-point Likert scale) | | | | Decision |
| --- | --- | --- | --- | --- | --- | --- | --- |
|  |  |  | Round #1 | | Round #2 | |  |
|  |  |  | Women | Clinicians | Women | Clinicians |  |
| 22 | Send patients information about their diagnosis of DCIS before the first physician visit so they can prepare questions | None | No consensus (64.7) | No consensus (20.0) | No consensus (43.8) | No consensus (31.6) | No consensus |
| 23 | Take extra time or schedule longer visits to discuss concerns and answer questions | None | Retain  (100.0) | No consensus (50.0) | Retain  (100.0) | No consensus (52.6) | No consensus |
| 24 | Ask patients about specific concerns | None | Retain  (100.0) | No consensus (70.0) | --- | --- | Retain  [combined rating of 83.8] |
| 25 | Use visual aids (pictures, models) to help explain DCIS | None | Retain  (88.2) | Retain  (80.0) | --- | --- | Retain |
| 26 | Use pathology or radiology report to supplement discussion | None | No consensus (74.5) | No consensus (55.0) | Retain  (87.5) | No consensus (73.7) | Retain  [combined rating of 80.0] |
| 27 | Provide physicians with visual aids or guides to help explain DCIS | None | Retain  (82.4) | Retain  (85.0) | --- | --- | Retain |
| 28 | Give physicians access to interpreters for patients with English as a second language | None | Retain  (88.2) | Retain  (90.0) | --- | --- | Retain |
| 29 | Provide patients with, or refer them to print or online resources about DCIS | None | No consensus (70.6) | No consensus (75.0) | Retain  (93.8) | Retain  (84.2) | Retain |
| 30 | Connect patients with services or groups for more information and support | None | Retain  (88.2) | No consensus (45.0) | Retain  (93.0) | No consensus (68.4) | Retain  [combined rating of 80.0] |
| 31 | Develop information for patients that is specific to DCIS (not included in resources about invasive breast cancer) | None | Retain  (94.1) | Retain  (80.0) | --- | --- | Retain |
| 32 | Develop information for patients about DCIS that is culturally tailored (e.g. available in different languages) | None | Retain  (88.2) | Retain  (80.0) | --- | --- | Retain |
| 33 | Arrange follow-up visit not long after first visit to discuss further concerns/ questions | Arrange follow-up visit (in-person or virtual) not long after first visit to discuss further concerns or questions | Retain  (88.2) | No consensus  (35.0) | Retain  (81.3) | No consensus (57.9) | No consensus |
| 34 | Do not manage low-risk DCIS in cancer centres to avoid giving patients the idea that they have full-blown cancer | Not favoured by panelists; not included in Round #2 survey | No consensus (29.4) | No consensus (15.0) | --- | --- | Discard |

Dissemination strategies

| Survey item | Label | Revision suggested in Round #1 and offered in Round #2 | Rating (% panelists who chose 6 or 7 on the 7-point Likert scale) | | | | Decision |
| --- | --- | --- | --- | --- | --- | --- | --- |
|  |  |  | Round #1 | | Round #2 | |  |
|  |  |  | Women | Clinicians | Women | Clinicians |  |
| 35 | Physicians should employ labels, language and other strategies identified by this research to decrease patient anxiety | None | Retain  (94.1) | Retain  (80.0) | --- | --- | Retain |
| 36 | Existing breast cancer public awareness campaigns and support groups should share information with women about DCIS | None | Retain  (88.2) | No consensus  (60.0) | Retain  (100.0) | Retain  (89.5) | Retain |
| 37 | Various types of organizations should provide continuing education for physicians (meetings and materials) about DCIS | None | Retain  (94.1) | No consensus  (50.0) | Retain  (100.0) | No consensus  (78.9) | Retain  [combined rating of 91.4] |
| 38 | Professional societies should share information with physicians about ideal labels, language and other strategies to improve communication about DCIS | None | Retain  (88.2) | No consensus  (65.0) | Retain  (100.0) | Retain  (84.2) | Retain |
| 39 | Organizations (e.g. professional, advocacy) should collaborate to establish widespread multidisciplinary consensus on ideal DCIS labels, language and other strategies to improve communication about DCIS | None | Retain  (88.2) | No consensus  (65.0) | Retain  (87.5) | No consensus  (78.9) | Retain  [combined rating of 82.9] |
| 40 | Change DCIS labels and language currently in medical records (now accessible to patients) to those identified in this research | Not favoured by panelists; not included in Round #2 survey | No consensus  (52.9) | No consensus (20.0) | --- | --- | Discard |
| 41 | Share the results of this research with cancer nomenclature agencies, which may influence processes and decisions about naming for low-risk DCIS | None | No consensus  (70.6) | No consensus (60.0) | Retain  (87.5) | No consensus  (63.2) | No consensus |
| 42 | Publish the results of this research in a prominent medical journal to encourage widespread use of ideal labels, language and other strategies | None | No consensus  (76.5) | No consensus (70.0) | Retain  (93.8) | No consensus  (73.7) | Retain  [combined rating of 82.9] |
