# Supplementary material for: Labels, Language, and Other Strategies to Improve Communication About Lower Grade Forms of Ductal Carcinoma In Situ of the Breast: A National Delphi Survey
Source: Int J Breast Cancer. 2025 Feb 17;2025:8642832. doi: 10.1155/ijbc/8642832 (PMC11850068; doi:10.1155/ijbc/8642832)
Supplement: Supporting Information 3 — File S3: Panelist comments about survey items. [file 8642832.f3.docx]

**Supplementary File 3. Panelist comments about survey items**

Preferred label(s) for DCIS

| Item | Label and Decision (percent of panelists who agreed or strongly agreed) | Comments | |
| --- | --- | --- | --- |
|  |  | Women | Clinicians |
| 1 | Abnormal cells of the breast duct that have not spread to breast tissue outside of the ducts  No consensus  (77.1) | PRO (Round #1)   - If DCIS is not cancer, as a patient, I would prefer it if the doctor said it was 'abnormal cells'. It is less scary than the other options. Can also explain that we want to do more surveillance in the future, etc. - I feel that it's important to use words that indicate abnormality in the cells, but not the word cancer, if the goal is indeed to reduce anxiety in patients. Once people hear the word "cancer" it's hard to hear that it's low risk of progressing or becoming invasive. These details can and should be explained as part of the education process. - The 'Pre-cancer' and 'abnormal cells' terms make a lot more sense.   PRO (Round #2)  My preference in communication of this type of DCIS to patients are #1 [abnormal cells] and #2 [pre-cancer], as they highlight the fact that cells are abnormal and have not spread and this is a pre-cancerous condition. This is different from calling it "cancer," which will trigger a great deal of anxiety.  CON (Round #1)  I do not buy into burying one's head in the sand to call cancer "abnormal cells" and not cancer because DCIS is cancer.  CON (Round #2)  Abnormal cells could be anything and could give patients a false sense of security, causing them to delay needed treatment | PRO (Round #1)   - Abnormal cells in the ducts of breast that have not spread to outside breast tissue - Abnormal cells that have not "learned the trick" to invade the tissue around it, which is how we label something as cancer. I therefore do not like terms that reference cancer.   PRO (Round #2)  ---  CON (Round #1)  Too vague and do not carry the gravity that is needed (1, 2, 3, 12) especially since those terms are more appropriate for true atypia e.g. ADH, ALH, etc.  CON (Round #2)  Option 1 is too wordy. Patients need clear, concise language. |
| 2 | Atypical cells of the breast duct  Discard  (29.7) | PRO (Round #1)  For some reason, the word atypical is less alarming than abnormal to me  CON (Round #1)  --- | PRO (Round #1)  ---  PRO (Round #2)  I would be happy if re-named "atypia" similar to cervical atypia etc. (and you take the word "cancer" out)  CON (Round #1)   - The cells are not dysplasia nor atypia, so we should not use those words - Too vague and does not carry the gravity that is needed (1, 2, 3, 12) especially since those terms are more appropriate for true atypia e.g. ADH, ALH, etc. |
| 3 | Breast duct dysplasia  Discard  (5.4) | PRO (Round #1)  ---  CON (Round #1)   - Neoplasia and dysplasia don't mean anything to me so if those words were in the name I may not understand what that meant - For questions 3 and 12 [Breast duct dysplasia and breast duct neoplasia], I gave the names a low rating because people may not have a clue what one of the words means so would not be well informed by that name. - The terms 'dysplasia' and 'neoplasia' are not common among non-medical people. I would avoid these confusing terms. - Some of the terms need to be explained to patients if they aren't familiar with medical terms (I.e. dysplasia, neoplasia, DCIS). | PRO (Round #1)  I would like to see a transition to dysplasia terminology.  CON (Round #1)   - The cells are not dysplasia nor atypia, so we should not use those words. - Too vague and does not carry the gravity that is needed (1, 2, 3, 12) especially since those terms are more appropriate for true atypia e.g. ADH, ALH, etc. |
| 4 | Pre-cancer  No consensus  (54.3) | PRO (Round #1)   - It's important to have people understand that DCIS is not Cancer and that in order to avoid the area becoming cancerous it needs to be treated. So the word Cancer Really shouldn't be used just to relieve some of the anxieties around being diagnosed with Cancer. You need to keep the language simple saying that the cells that they found are pre-cancerous and can potentially become cancerous, but they are not cancerous. - The 'Pre-cancer' and 'abnormal cells' terms make a lot more sense.   PRO (Round #2)  The ability to distinguish types of breast cancers is important, so I agree with keeping the name, provided it's qualified with pre or stage 0  CON (Round #1)   - I think providers should be careful about using the term pre-cancer, which is misleading as in DCIS, cells are malignant. - #4 [pre-cancer] feels undefined - I strongly disagree with calling it pre-cancer because the impacts of treatments mentally and physically is the same as cancer.   CON (Round #2)  --- | PRO (Round #1)   - Pre-cancer is more aligned with my philosophy however I caution patients that we do not yet know which DCIS would have later started to invade (and therefore be called cancer) - I don't like the labels that include the word cancer or carcinoma. I think it is confusing to patients. Though I do use the term pre-cancer in my practice   PRO (Round #2)  The word "cancer" is scary, but "pre-cancer" can be understood by all.  CON (Round #1)  I don't like the word "precursor" as it is not a word used in regular speech, and would not be familiar to people who have English as a second language.  CON (Round #2)  Re: Pre-cancer, stage 0 cancer - Using the word "cancer" to describe DCIS is incorrect, as DCIS is missing the "invasive" component of definition of a cancer. I think that it is easier for a patient to understand the treatment of DCIS if they understand/accept it is not cancer, and so treatment aims at local control, and not concern of distant relapse. As our management of low-grade DCIS evolves, and foreseeably we might not treat all lesions with surgery in the future, I speculate it could be difficult for patients to reconcile/accept a "cancer" being left in the breast. |
| 5 | Stage 0 breast cancer  No consensus  (54.3) | PRO (Round #1)   - I think it should be named and considered as Cancer as it needs to follow the cancer treatments like radiotherapy, medicine therapy and if you ignore the treatments and don't count it seriously it can lead to invasive breast cancer. - #5 [stage 0 breast cancer] at least tells me where I sit on the scale   PRO (Round #2)  The ability to distinguish types of breast cancers is important, so I agree with keeping the name, provided it's qualified with pre or stage 0  CON (Round #1)  Using the term 'cancer' when it isn't yet actual cancer is extremely confusing.  CON (ROUND #2)  --- | PRO (Round #1)  Assuming these are the labels for patient facing communication and medical label of DCIS/stage 0 remains? It is important to maintain consistency in medical reporting for research/cancer statistics reporting  PRO (Round #2)  ---  CON (Round #1)   - Any label with "cancer" in the wording will spark naturally more concern in patients. - "Breast cancer" is usually understood as invasive breast carcinoma. This term should not be used for DCIS unless accompanied by adjective indicating it is non-invasive. - I don't like the labels that include the word cancer or carcinoma. - I think when a physicians says cancer, 99% of the time, whether they know it or not, they are referring to "invasive cancer". Non-invasive cancers are the exception, not the rule. Characterizing DCIS as a "cancer" of any form I think muddies the water. It is easy enough to explain that things that are not currently cancer can lead to cancer.   CON (Round #2)   - RE: pre-cancer, stage 0 cancer - Using the word "cancer" to describe DCIS is incorrect, as DCIS is missing the "invasive" component of definition of a cancer. I think that it is easier for a patient to understand the treatment of DCIS if they understand/accept it is not cancer, and so treatment aims at local control, and not concern of distant relapse. As our management of low-grade DCIS evolves, and foreseeably we might not treat all lesions with surgery in the future, I speculate it could be difficult for patients to reconcile/accept a "cancer" being left in the breast. - I don't think "cancer" or "carcinoma" should be in the title at all. For these LOWER GRADE forms of DCIS, most will NEVER progress to invasive disease in a woman's lifespan |
| 6 | Pre-invasive breast cancer  Discard  (29.7) | PRO (Round #1)  I think it should be named and considered as Cancer as it needs to follow the cancer treatments like radiotherapy, medicine therapy and if you ignore the treatments and don't count it seriously it can lead to invasive breast cancer.  CON (Round #1)   - Using the term 'cancer' when it isn't yet actual cancer is extremely confusing. - If DCIS is not invasive, why would this label be used? | PRO (Round #1)  ---  CON (Round #1)   - Any label with "cancer" in the wording will spark naturally more concern in patients. - I don't like the labels that include the word cancer or carcinoma. - "Breast cancer" is usually understood as invasive breast carcinoma. This term should not be used for DCIS unless accompanied by adjective indicating it is non-invasive. - I think when a physicians says cancer, 99% of the time, whether they know it or not, they are referring to "invasive cancer". Non-invasive cancers are the exception, not the rule. Characterizing DCIS as a "cancer" of any form I think muddies the water. It is easy enough to explain that things that are not currently cancer can lead to cancer. |
| 7 | Early-stage breast cancer  Discard  (18.9) | PRO (Round #1)  I think it should be named and considered as Cancer as it needs to follow the cancer treatments like radiotherapy, medicine therapy and if you ignore the treatments and don't count it seriously it can lead to invasive breast cancer.  CON (Round #1)   - Using the term 'cancer' when it isn't yet actual cancer is extremely confusing. - #14 [early form of breast cancer] and #7 [early-stage breast cancer] - These also sound like it could become something else - like this is just the beginning stage, as opposed to a low-risk stage. I notice that most of these still use the word cancer. I am a bit confused because the consent form says that DCIS is not cancer...and yet the word remains. I was told it was cancer - low risk, stage 0, but cancer, nevertheless. I think it might be hard to explain why a woman needs surgery/treatment without using that word. Your information sheet is clearer - it suggests the confusion is around invasive vs. non-invasive - and I think sorting that out is an important goal! | PRO (Round #1)  ---  CON (Round #1)   - Any label with "cancer" in the wording will spark naturally more concern in patients. - I don't like the labels that include the word cancer or carcinoma. - "Breast cancer" is usually understood as invasive breast carcinoma. This term should not be used for DCIS unless accompanied by adjective indicating it is non-invasive. - I think when a physicians says cancer, 99% of the time, whether they know it or not, they are referring to "invasive cancer". Non-invasive cancers are the exception, not the rule. Characterizing DCIS as a "cancer" of any form I think muddies the water. It is easy enough to explain that things that are not currently cancer can lead to cancer. |
| 8 | Ductal carcinoma in situ  No consensus  (42.9) | PRO (Round #1)  Isn't this what DCIS stands for? So I like this name because that is, in fact (as I understand it) what is going on in the body.  PRO (Round #2)  ---  CON (Round #1)  Some of the terms need to be explained to patients if they aren't familiar with medical terms (I.e. dysplasia, neoplasia, DCIS).  CON (Round #2)  --- | PRO (Round #1)  Assuming these are the labels for patient facing communication and medical label of DCIS/stage 0 remains? It is important to maintain consistency in medical reporting for research/cancer statistics reporting  PRO (Round #2)  ---  CON (Round #1)   - I don't like labels that include the word cancer or carcinoma. I think it is confusing to patients. - DCIS -- is currently used in pathology reports and media, and patients often have access to their path reports. So, this "official term" needs to be addressed and explained, at least for now.   CON (Round #2)  I don't think "cancer" or "carcinoma" should be in the title at all. For these LOWER GRADE forms of DCIS, most will NEVER progress to invasive disease in a woman's lifespan |
| 9 | Low-risk breast cancer  Discard  (21.6) | PRO (Round #1)  I think it should be named and considered as Cancer as it needs to follow the cancer treatments like radiotherapy, medicine therapy and if you ignore the treatments and don't count it seriously it can lead to invasive breast cancer.  CON (Round #1)  Using the term 'cancer' when it isn't yet actual cancer is extremely confusing. | PRO (Round #1)  ---  CON (Round #1)   - Any label with "cancer" in the wording will spark naturally more concern in patients. - I don't like the labels that include the word cancer or carcinoma. - "Breast cancer" is usually understood as invasive breast carcinoma. This term should not be used for DCIS unless accompanied by adjective indicating it is non-invasive. - I think when a physicians says cancer, 99% of the time, whether they know it or not, they are referring to "invasive cancer". Non-invasive cancers are the exception, not the rule. Characterizing DCIS as a "cancer" of any form I think muddies the water. It is easy enough to explain that things that are not currently cancer can lead to cancer. - I therefore do not like terms that reference cancer (eg. 9-11, 13, 14) |
| 10 | Low-grade breast cancer  Discard  (10.8) | PRO (Round #1)  I think it should be named and considered as Cancer as it needs to follow the cancer treatments like radiotherapy, medicine therapy and if you ignore the treatments and don't count it seriously it can lead to invasive breast cancer.  CON (Round #1)  Using the term 'cancer' when it isn't yet actual cancer is extremely confusing. | PRO (Round #1)  ---  CON (Round #1)   - Any label with "cancer" in the wording will spark naturally more concern in patients. - I don't like the labels that include the word cancer or carcinoma. - "Breast cancer" is usually understood as invasive breast carcinoma. This term should not be used for DCIS unless accompanied by adjective indicating it is non-invasive. - I use low grade and non-aggressive to talk about invasive disease the early stage too. - I think when a physicians says cancer, 99% of the time, whether they know it or not, they are referring to "invasive cancer". Non-invasive cancers are the exception, not the rule. Characterizing DCIS as a "cancer" of any form I think muddies the water. It is easy enough to explain that things that are not currently cancer can lead to cancer. - I therefore do not like terms that reference cancer (eg. 9-11, 13, 14) |
| 11 | Non-aggressive breast cancer  Discard  (5.4) | PRO (Round #1)  I think it should be named and considered as Cancer as it needs to follow the cancer treatments like radiotherapy, medicine therapy and if you ignore the treatments and don't count it seriously it can lead to invasive breast cancer.  CON (Round #1)  Using the term 'cancer' when it isn't yet actual cancer is extremely confusing. | PRO (Round #1)  ---  CON (Round #1)   - Any label with "cancer" in the wording will spark naturally more concern in patients. - I don't like the labels that include the word cancer or carcinoma. - "Breast cancer" is usually understood as invasive breast carcinoma. This term should not be used for DCIS unless accompanied by adjective indicating it is non-invasive. - I use low grade and non-aggressive to talk about invasive disease the early stage too. - I think when a physicians says cancer, 99% of the time, whether they know it or not, they are referring to "invasive cancer". Non-invasive cancers are the exception, not the rule. Characterizing DCIS as a "cancer" of any form I think muddies the water. It is easy enough to explain that things that are not currently cancer can lead to cancer. I therefore do not like terms that reference cancer (eg. 9-11, 13, 14) |
| 12 | Breast duct neoplasia  Discard  (2.7) | PRO (Round #1)  ---  CON (Round #1)   - Neoplasia and dysplasia don't mean anything to me so if those words were in the name I may not understand what that meant - For questions 3 and 12 [Breast duct dysplasia and breast duct neoplasia], I gave the names a low rating because people may not have a clue what one of the words means so would not be well informed by that name. - #3 #12- I would need to look up 'dysplasia' and 'neoplasia' as I have no idea what those words mean. Not too lay friendly! - The terms 'dysplasia' and 'neoplasia' are not common among non-medical people. I would avoid these confusing terms. - Some of the terms need to be explained to patients if they aren't familiar with medical terms (I.e. dysplasia, neoplasia, DCIS). | PRO (Round #1)  ---  CON (Round #1)   - Neoplasm is so broad rendering it useless - Too vague and does not carry the gravity that is needed (1, 2, 3, 12) especially since those terms are more appropriate for true atypia e.g. ADH, ALH, etc. |
| 13 | Non-invasive breast cancer  Discard  (18.9) | PRO (Round #1)  I think it should be named and considered as Cancer as it needs to follow the cancer treatments like radiotherapy, medicine therapy and if you ignore the treatments and don't count it seriously it can lead to invasive breast cancer.  CON (Round #1)   - It is helpful that it is called non-invasive, but this name doesn't relay that this is low risk. It sounds like full-on cancer - Using the term 'cancer' when it isn't yet actual cancer is extremely confusing. | PRO (Round #1)  ---  CON (Round #1)   - Any label with "cancer" in the wording will spark naturally more concern in patients. - I don't like the labels that include the word cancer or carcinoma. - "Breast cancer" is usually understood as invasive breast carcinoma. This term should not be used for DCIS unless accompanied by adjective indicating it is non-invasive. - I think when a physicians says cancer, 99% of the time, whether they know it or not, they are referring to "invasive cancer". Non-invasive cancers are the exception, not the rule. Characterizing DCIS as a "cancer" of any form I think muddies the water. It is easy enough to explain that things that are not currently cancer can lead to cancer. - I therefore do not like terms that reference cancer (eg. 9-11, 13, 14) |
| 14 | Early form of breast cancer  Discard  (13.5) | PRO (Round #1)  I think it should be named and considered as Cancer as it needs to follow the cancer treatments like radiotherapy, medicine therapy and if you ignore the treatments and don't count it seriously it can lead to invasive breast cancer.  CON (Round #1)   - Using the term 'cancer' when it isn't yet actual cancer is extremely confusing. - #14 [early form of breast cancer] and #7 [early stage breast cancer] - These also sound like it could become something else - like this is just the beginning stage, as opposed to a low-risk stage. I notice that most of these still use the word cancer. I am a bit confused because the consent form says that DCIS is not cancer...and yet the word remains. I was told it was cancer - low risk, stage 0, but cancer nevertheless. I think it might be hard to explain why a woman needs surgery/treatment without using that word. Your information sheet is more clear - it suggests the confusion is around invasive vs. non-invasive - and I think sorting that out is an important goal! | PRO (Round #1)  ---  CON (Round #1)   - Any label with "cancer" in the wording will spark naturally more concern in patients. - I don't like the labels that include the word cancer or carcinoma. - "Breast cancer" is usually understood as invasive breast carcinoma. This term should not be used for DCIS unless accompanied by adjective indicating it is non-invasive. - I think when a physicians says cancer, 99% of the time, whether they know it or not, they are referring to "invasive cancer". Non-invasive cancers are the exception, not the rule. Characterizing DCIS as a "cancer" of any form I think muddies the water. It is easy enough to explain that things that are not currently cancer can lead to cancer. - I therefore do not like terms that reference cancer (eg. 9-11, 13, 14) |

Language to explain DCIS

| Item | Strategy and Decision (percent of panelists who agreed or strongly agreed) | Comments | |
| --- | --- | --- | --- |
|  |  | Women | Clinicians |
| 15 | Use plain/lay  language to explain DCIS  Retain  (89.1) | PRO (Round #1)  When explaining (again in lay person terms) it is recommended that the explanation then be related to what has been diagnosed for that patient, so they are dealing with only the  information pertaining to them.  CON (Round #1)  --- | PRO (Round #1)  ---  CON (Round #1)  Also, defining "lay" language is difficult -- a physician's "lay" language may indeed be quite advanced for a patient. Sometimes it is hard to gauge in real life. |
| 16 | State that DCIS is not invasive breast cancer because it stays in the breast duct and is unlikely to spread  Retain  (80.0) | PRO (Round #1)  ---  PRO (Round #2)  ---  CON (Round #1)  I agree with the first part that DCIS is not invasive; the second part is a blanket statement and a future prediction. you cannot assume that DCIS will stay in the breast and will not spread. what you could say instead is DCIS is the earliest stage of breast cancer which has not spread outside of the milk ducts in the breast. the risk of DCIS recurrence or invasion depends on the grade of DCIS and tumor characteristics.  CON (Round #2)  What percentage is DCIS NOT likely to spread? | PRO (Round #1)  ---  PRO (Round #2)   - DCIS - atypical cells which are only within the milk ducts in the breast. About half the time these cells will cause no issue, but about half of cases have the potential to evolve into invasive breast cancer over time. - State that DCIS is not invasive breast cancer because it IS CURRENTLY in the breast duct. ALSO INDICATE THAT IT is unlikely to spread.   CON (Round #1)   - I disagree with (#16) as we do not presently know which DCIS will "stay in the breast duct and be unlikely to spread". I agree it is not presently invasive breast cancer. - Most patients ask about the risk of becoming cancer or spread. So, I think this needs to be addressed. Also justifies the treatments.   CON (Round #2)   - Currently there is no way to know what DCIS is "going to do" in any given patient case so I do not endorse that language, may be falsely reassuring; can only speak about the current diagnosis and that there is no present sign of spread/cancer - DCIS "stays in the breast duct and is unlikely to spread" has some potential trouble for interpretation as it can become invasive over time if not removed, and then it could spread. - DCIS does not invade the body, but if left untreated may later transform into real cancer. I recommend treatment now to prevent this transformation." |
| 17 | Use analogies  to explain DCIS  No consensus  (42.9) | PRO (Round #1)  ---  PRO (Round #2)  ---  CON (Round #1)  ---  CON (Round #2)  --- | PRO (Round #1)  ---  PRO (Round #2)  ---  CON (Round #1)  Not sure what "analogies" means exactly -- could be helpful or not, depending on the situation given and the patient's understanding. May lead to further confusion, especially if English not first language.  CON (Round #2)  Analogies -- may confuse even more. It would depend on the patient and the analogy. |
| 18 | Mention that DCIS affects many women  No consensus  (34.3) | PRO (Round #1)  ---  PRO (Round #2)  ---  CON (Round #1)  Does it matter that DCIS is common? Unless there is context, I don't know if this is helpful. In other words, it doesn't matter if it is common if everyone has a recurrence, because then it still sucks to have it. It would matter if it is common and doesn't generally turn into invasive breast cancer.  CON (Round #2)  Each patient should be treated as an individual, mentioning that we are one of many may not be helpful | PRO (Round #1)  ---  PRO (Round #2)  ---  CON (Round #1)  DCIS results in a referral to the cancer clinic. It is not "very common", that would seem to imply a high number of the population has DCIS.  CON (Round #2)  I am not sure what qualifies as "many women" and incidence depends on age |
| 19 | Explain DCIS by grade, where higher-grade forms of DCIS require treatment, and low-grade forms may not  No consensus  (34.3) | PRO (Round #1)  ---  PRO (Round #2)  ---  CON (Round #1)  My surgeon explained that this is now known to be old information, and that new research shows that all DCIS needs treatment to prevent it becoming invasive.  CON (Round #2)  My surgeon explained that DCIS used to be thought of as not cancer and didn't always need treatment, but modern research has shown that it actually is cancer and should always be treated because it will eventually become invasive | PRO (Round #1)  ---  PRO (Round #2)  ---  CON (Round #1)   - Spectrum is also a word not used in common conversation; I would not use "spectrum" for anyone with ESL. - I am not clear as to this survey's use of the phrase "Low risk DCIS". There is no accepted definition of "Low risk DCIS" that I am aware of. Is this referring to "Low grade DCIS"? - Important to qualify molecular subtype implications of DCIS- i.e. Triple negative DCIS more concerning than ER+ DCIS. -unclear what you mean by Q20- typically all DCIS is treated as we are not clear which DCIS will progress and which are not -important things we can use to determine "risk" levels of DCIS i.e Oncotype Dx, molecular subtypes which can help determine which needs treatment and which does not. -important to include specific % from literature re: rates of progression of untreated DCIS to invasive disease.   CON (Round #2)   - Many women cannot differentiate/confuse grade and stage when they hear or read it -although these are obviously very different. Using grade to explain may confuse the issue even more. - I'm not sure there is good data yet to support low grade forms don't require any treatment - Current standard of care is to treat all DCIS regardless of grade |
| 20 | Address risks (e.g. spread, recurrence)  and outcomes (e.g. prognosis) associated with low-risk DCIS  Retain  (83.8) | PRO (Round #1)  The MORE communication, the BETTER, even VERY UNLIKELY to be cancerous or malignant. Percentage unlikely will be helpful too. If I hear 99% chance not likely, qualify the statement vs just saying LIKELY/UNLIKELY..... that's subjective  CON (Round #1)  --- | PRO (Round #1)  ---  CON (Round #1)  --- |
| 21 | Discuss risk based  on stage or grade to explain why treatment is recommended for low-risk DCIS  Discard  (67.6) | PRO (Round #1) I wish I had been given more information on grade and chance of reoccurrence with and without treatment  CON (Round #1)  --- | PRO (Round #1)  ---  CON (Round #1)  DCIS is not cancer and therefore it is not appropriate to use "stage" to help explain risk. Grade is useful to describe how abnormal the  cells look under microscope, on a spectrum. Also many patients have copies of their path reports and ask about this anyway, in my experience. |

Other strategies to help explain DCIS

| Item | Strategy and Decision (percent of panelists who agreed or strongly agreed) | Comments | |
| --- | --- | --- | --- |
|  |  | Women | Clinicians |
| 22 | Send patients information about their diagnosis of DCIS before the first physician visit so they can prepare questions  No consensus  (37.1) | PRO (Round #1)  Give patients information about what to expect at visits. When I was in the waiting room for my biopsy I was scared because I had no idea what to expect. I remember thinking, "This is routine for medical staff, but for ME it's all new. Here I am waiting all alone with no clue about what is about to happen, what will be done to me, where I need to go, or any other details. Heck, I don't even know what the room looks like, never mind what will happen." I found that to be the case at every stage along the way.  PRO (Round #2)  ---  CON (Round #1)   - Sometimes providing information up front can create anxiety when patients try to interpret results in the absence of a doctor. Give access at time of appointment or just before, but allow enough time for the appointment so patients can ask questions - For some people, knowing ahead of time causes significant alarm, while for others it is a great chance to do some research.   CON (Round #2)  Sending information before the appointment may create more anxiety than necessary. | PRO (Round #1)  ---  PRO (Round #2)  ---  CON (Round #1)  ---  CON (Round #2)  --- |
| 23 | Take extra time or schedule longer visits to discuss concerns and answer questions  No consensus  (74.3) | PRO (Round #1)  ---  PRO (Round #2)  ---  CON (Round #1)  ---  CON (Round #2)  --- | PRO (Round #1)   - Consults for DCIS can often take longer than consults for true IMC in my experience because of the need to convey more complex risk information and ensure comprehension - My DCIS conversations take considerably longer than my conversations with patients with frankly far worse and riskier disease. I think it's because I provide stronger recommendations for riskier disease. Patients seem to find weak recommendations more difficult to navigate. (I find the evidence for adjuvant hormone therapy for DCIS underwhelming.)   PRO (Round #2)  I find that patients with DCIS need just as long as cancer patients in clinic - so they would be booked for same amount of time  CON (Round #1)  ---  CON (Round #2)   - There is limited time for physicians available to agonize with patients over DCIS treatment decisions. They can understand it is not invasive cancer, but then with multiple options to pick from for treatment or no treatment, it is very hard for those that can't make decisions. And the literature varies a lot - Extra time may be difficult to manage logistically in today's health care system |
| 24 | Ask patients about specific concerns  Retain  (83.8) | PRO (Round #1)  ---  CON (Round #1)  --- | PRO (Round #1)  ---  CON (Round #1)  --- |
| 25 | Use visual aids (pictures, models) to help explain DCIS  Retain  (83.8) | PRO (Round #1)  ---  PRO (Round #2)  ---  CON (Round #1)  ---  CON (Round #2)  --- | PRO (Round #1)  ---  PRO (Round #2)  Use 2 x 2 table: benefits & risks of treating, benefits & risks of not treating.  CON (Round #1)  ---  CON (Round #2)  --- |
| 26 | Use pathology or radiology report to supplement discussion  Retain  (80.0) | PRO (Round #1)  ---  PRO (Round #2)  ---  CON (Round #1)  ---  CON (Round #2)  --- | PRO (Round #1)  ---  PRO (Round #2)  ---  CON (Round #1)  ---  CON (Round #2)   - Using and radiology and path reports introduces yet MORE terminology that can be confusing. Our job as clinicians is to synthesize the reports, and present in digestible appropriate format based on the individual patient. - Pathology reports not helpful as they talk about grading, "comedonecrosis" gets everyone worried and then IHC stains get pts even more confused as if ER is negative, or Her2 done and positive, then patients read that they need chemo and have a bad prognosis. |
| 27 | Provide physicians with visual aids or guides to help explain DCIS  Retain  (83.8) | PRO (Round #1)  ---  PRO (Round #2)  ---  CON (Round #1)  ---  CON (Round #2)  --- | PRO (Round #1)  ---  PRO (Round #2)  ---  CON (Round #1)  ---  CON (Round #2)  --- |
| 28 | Give physicians access to interpreters for patients with English as a second language  Retain  (89.2) | PRO (Round #1)  I think it is critical to have information in different languages - Canada is multicultural.  CON (Round #1)  --- | PRO (Round #1)  ---  CON (Round #1)  --- |
| 29 | Provide patients with, or refer them to print or online resources about DCIS  Retain  (88.6) | PRO (Round #1)  I also think more information about DCIS (current information) is helpful - especially to take home so that we can read about it after the appointment.  PRO (Round #2)  Having access to follow up visits (14), resources (12) and services (13) for additional information will be helpful to those seeking additional support, but only if those are aligned in how they communicate about DCIS.  CON (Round #1)  Giving patients too much information at the beginning that they have to research may lead them down a negative hole. Maybe provide only specific reputable websites ONLY that will help support the information provided.  CON (Round #2)  Not all patients are tech savvy, especially seniors, and may not have the resources to access this info. For example, I live in a seniors building with 76 units and am one of only 3 or 4 residents who know how to use a cell phone or computer. | PRO (Round #1)  ---  PRO (Round #2)  There are not great resources for DCIS to refer patients to. Resources that incorporated diagrams of milk ducts, mammogram picture of microcalcifications helpful.  CON (Round #1)  ---  CON (Round #2)  The breast surgeon is responsible for explaining DCIS to his/her patient in a language appropriate for her. Other resources may be confusing and contradict what the surgeon said. |
| 30 | Connect patients with services or groups for more information and support  Retain  (80.0) | PRO (Round #1)  ---  PRO (Round #2)  Having access to follow up visits (14), resources (12) and services (13) for additional information will be helpful to those seeking additional support, but only if those are aligned in how they communicate about DCIS.  CON (Round #1)  ---  CON (Round #2)  --- | PRO (Round #1)  ---  PRO (Round #2)  ---  CON (Round #1)  ---  CON (Round #2)  --- |
| 31 | Develop information for patients that is specific to DCIS (not included in resources about invasive breast cancer)  Retain  (86.5) | PRO (Round #1)  ---  CON (Round #1)  --- | PRO (Round #1)  DCIS-specific information without cancer language would be welcomed.  CON (Round #1)  --- |
| 32 | Develop information  for patients about DCIS that is culturally tailored (e.g. available in different languages)  Retain  (83.8) | PRO (Round #1)  I think it is critical to have information in different languages - Canada is multicultural.  CON (Round #1)  --- | PRO  All patients deserve access to information in their language of choice, reviewed to be culturally relevant.  CON (Round #1)  --- |
| 33 | Arrange follow-up visit not long after first visit to discuss further concerns/ questions  No consensus  (68.6) | PRO (Round #1)   - Follow-up visits could be virtually to reduce the angst of going to the hospital. - I think, in general, it would be great to offer #33 [follow-up visits] because I am sure it is common to leave the first appointment and think of 6 more questions.   PRO (Round #2)   - Setting two appointments, close together allows a patient to develop questions after the first appointment instead of receiving their diagnosis in absence of a doctor - Having access to follow up visits (14), resources (12) and services (13) for additional information will be helpful to those seeking additional support, but only if those are aligned in how they communicate about DCIS.   CON (Round #1)  ---  CON (Round #2)  --- | PRO (Round #1)  As patient I would appreciate the opportunity however may not be the best use of resources. Perhaps an opportunity for a call if additional questions.  PRO (Round #2)  ---  CON (Round #1)  ---  CON (Round #2)  --- |
| 34 | Do not manage low-risk DCIS in cancer centres to avoid giving patients the idea that they have full-blown cancer  Discard  (21.6) | PRO (Round #1)  #34 makes a lot of sense, if it's truly just a pre-cancer then treat it as such.  CON (Round #1)  I am biased - I work in a cancer support centre, so I think providing information for that kind of support is critical. I think being treated in a cancer centre is important. Since the surgery and treatments can mirror those of invasive cancers, it might be more difficult to believe that outside support is needed - why should I be feeling this way when I don't have cancer? And even if DCIS is low risk, stage zero - the concern is that it could worsen, as I understand it - so being treated in a cancer centre might give the person with DCIS a sense of comfort - as in, I am being treated by those who know cancer. | PRO (Round #1)  ---  CON (Round #1)   - How do you define "low-risk DCIS" Depends what treatments they will need as to where they are managed. - Some of these may be logistically impossible (e.g. having clinics at a different location for these patients, translation) - We do not have community resources available to counsel pts and to provide DCIS management outside of the cancer clinics. |

Dissemination approaches to promote use of study findings

| Item | Strategy and Decision (percent of panelists who agreed or strongly agreed) | Comments | |
| --- | --- | --- | --- |
|  |  | Women | Clinicians |
| 35 | Physicians should  employ labels, language and other strategies identified by this research to decrease patient anxiety  Retain  (86.5) | PRO (Round #1)   - Provided the labels and communications used are grounded in scientific evidence and are not superficially changed with the only intent of reducing patient anxiety (the road to good intentions....] - If label/name changes, future diagnosis should make reference to the fact that "this was previously referred to DCIS"   CON (Round #1)  --- | PRO (Round #1)  ---  CON (Round #1)  We should not rebrand and change the pathology nomenclature for DCIS. Then it becomes confusing, “the condition formerly known as DCIS" aka Prince or Twitter...That makes things more confusing or patients doing the research. The previous efforts in BC to relabel things as DIN1 etc did not catch on, and were just confusing for both physicians and patients. We should not stop calling them cancer cells inside the milk duct, just to make some patients feel less anxious about a diagnosis. We should provide the education to reduce the anxiety. Also, the local recurrence risk needs to be considered, if we trivialize it into "nothing" then we will have a significant proportion of patients who will eventually need more biopsies and surgeries later in life. Having to think about potential for additional biopsies and surgeries later in life also does not help patient well-being. |
| 36 | Existing breast cancer public awareness campaigns and support groups should share information with women about DCIS  Retain  (94.3) | PRO (Round #1)  ---  PRO (Round #2)  ---  CON (Round #1)  ---  CON (Round #2)  --- | PRO (Round #1)  ---  PRO (Round #2)  ---  CON (Round #1)  ---  CON (Round #2)  --- |
| 37 | Various types  of organizations should provide continuing education for physicians (meetings and materials) about DCIS  Retain  (88.6) | PRO (Round #1)  ---  PRO (Round #2)  ---  CON (Round #1)  ---  CON (Round #2)  --- | PRO (Round #1)  ---  PRO (Round #2)  ---  CON (Round #1)  ---  CON (Round #2)  --- |
| 38 | Professional societies  should share information with physicians about ideal labels, language and other strategies to improve communication about DCIS  Retain  (91.4) | PRO (Round #1)  ---  PRO (Round #2)  ---  CON (Round #1)  ---  CON (Round #2)  --- | PRO (Round #1)  ---  PRO (Round #2)  ---  CON (Round #1)  ---  CON (Round #2)  --- |
| 39 | Organizations (e.g. professional, advocacy) should collaborate to establish widespread multidisciplinary consensus on ideal DCIS labels, language and other strategies to improve communication about DCIS  Retain  (82.9) | PRO (Round #1)  ---  PRO (Round #2)  ---  CON (Round #1)  ---  CON (Round #2)  --- | PRO (Round #1)  ---  PRO (Round #2)  ---  CON (Round #1)  ---  CON (Round #2)  --- |
| 40 | Change DCIS labels and language currently in medical records (now accessible to patients) to those identified in this research  Discard  (35.1) | PRO (Round #1)  ---  CON (Round #1)  I am STRONGLY opposed to changing what is in my health record. I have lived through DCIS twice. Once I asked my surgeon about how I had seen, here and there, that I didn't have 'real' cancer and so I was upset. She said that I had surgery, treatment, tamoxifen, more surgery...I had cancer. Low risk, stage 0...but cancer. My records says breast cancer. I have done a ton of emotional work to live with this and the changes to my body. I think it would be very hard on me to have that terminology changed because I have come to terms with it, and see myself as a survivor. That's just one woman's opinion. It is different if this is a first diagnosis...but I really really wouldn't want it changed on my history | PRO (Round #1)  ---  CON (Round #1)   - Medical labels need to remain in order to allow ongoing research and clear registry of cases. Without this, the research which ultimately informs risk and permits de-escalation or escalation of treatment will be hampered. - Don't know how you would do this - Medical records cannot be changed retrospectively - Pathologists are not free to change the name of tumours, but may add a standardized risk score associated with this lesion (similar to radiologist BI-RADS system) |
| 41 | Share the results  of this research with cancer nomenclature agencies, which may influence processes and decisions about naming for low-risk DCIS  No consensus  (74.3) | PRO (Round #1)  ---  PRO (Round #2)  ---  CON (Round #1)  ---  CON (Round #2)  --- | PRO (Round #1)  ---  PRO (Round #2)  ---  CON (Round #1)   - I don't think without widespread agreement from professional societies - we can change labels. Tools for explaining DCIS would be useful. - While consistent language and nomenclature is important, engaging too many agencies will only serve to delay real time change - We should not rebrand and change the pathology nomenclature for DCIS. Then it becomes confusing, "the condition formerly known as DCIS" aka Prince or Twitter...That makes things more confusing or patients doing the research. The previous efforts in BC to relabel things as DIN1 etc did not catch on, and were just confusing for both physicians and patients. We should not stop calling them cancer cells inside the milk duct, just to make some patients feel less anxious about a diagnosis. We should provide the education to reduce the anxiety. Also the local recurrence risk needs to be considered, if we trivialize it into "nothing" then we will have a significant proportion of patients who will eventually need more biopsies and surgeries later in life. Having to think about potential for additional biopsies and surgeries later in life also does not help patient well-being.   CON (Round #2)   - Everyone should use the same language. Unfortunately, clinicians are in conflict of interest. They will prefer to sound worried to increase procedures and billing. - We already tried to change DCIS to DIN classification, which became more confusing for everyone. Let's not rebrand DCIS but just provide more specific educational resources. There is also a lot of variation in physician opinions about de-escalation strategy. The de-escalation comes with increased potential for more biopsies and surgeries down the road, despite reduced treatment at the beginning. |
| 42 | Publish the results  of this research in a prominent medical journal to encourage widespread use of ideal labels, language and other strategies  Retain  (82.9) | PRO (Round #1)  ---  PRO (Round #2)  ---  CON (Round #1)  ---  CON (Round #2)  --- | PRO (Round #1)  ---  PRO (Round #2)  ---  CON (Round #1)  ---  CON (Round #2)  --- |
